# Novel Variants of a Histogram Shift-Based Reversible Watermarking Technique for Medical Images to Improve Hiding Capacity

**DOI:** 10.1155/2017/3538979

**Published:** 2017-09-26

**Authors:** Vishakha Kelkar, Kushal Tuckley, Hitesh Nemade

**Affiliations:** ^1^UMIT, SNDT University, Mumbai, Maharashtra, India; ^2^AGV Systems Pvt. Ltd. India, Mumbai, Maharashtra, India; ^3^D.J. Sanghvi Engineering College, Mumbai, Maharashtra, India

## Abstract

In telemedicine systems, critical medical data is shared on a public communication channel. This increases the risk of unauthorised access to patient's information. This underlines the importance of secrecy and authentication for the medical data. This paper presents two innovative variations of classical histogram shift methods to increase the hiding capacity. The first technique divides the image into nonoverlapping blocks and embeds the watermark individually using the histogram method. The second method separates the region of interest and embeds the watermark only in the region of noninterest. This approach preserves the medical information intact. This method finds its use in critical medical cases. The high PSNR (above 45 dB) obtained for both techniques indicates imperceptibility of the approaches. Experimental results illustrate superiority of the proposed approaches when compared with other methods based on histogram shifting techniques. These techniques improve embedding capacity by 5–15% depending on the image type, without affecting the quality of the watermarked image. Both techniques also enable lossless reconstruction of the watermark and the host medical image. A higher embedding capacity makes the proposed approaches attractive for medical image watermarking applications without compromising the quality of the image.

## 1. Introduction

Rapid growth in the internet and multimedia technology in the recent times poses threat to the authentication and secured transmission of multimedia data. In telemedicine applications, the medical images are exchanged to facilitate improved patient's clinical status [1]. As the communication channel is shared between multiple users, poor security services may have adverse effect on the quality of care provided. The quality of biomedical images needs to be strictly maintained. The presence of an artefact and other impurities in the medical data may cause diagnostic errors [2]. Patient's information security is crucial to support the trust relationship. So, watermarking techniques are used for authentication. However, watermarking can cause harm to the sensitive information present in the cover image. In medical imagery, a small change in the cover image may affect interpretation significantly. Consequently, the watermarking methods used for medical applications must be reversible, in which the original medical image must be recovered pixel by pixel accurately. Most reversible watermarking methods impose limitation on the hiding capacity. Another approach for using watermarking for medical images could be to keep intact the region of interest and to embed the watermark in the region of noninterest [3].

Various algorithms have been proposed by various researchers for reversible watermarking. These techniques can be categorised in to five classes, namely, (i) integer transform based, (ii) data compression based, (iii) based on histogram bin shifting, (iv) prediction of pixel values based, and (v) based on modification of frequency domain characteristics [4].

The histogram bin shifting-based methods are popular because of the ease of implementation and the overhead generated is lesser. It makes use of the histogram of the original image, modifies selected portion of the histogram near the peak point, and then embeds the secret data into the image. The scheme was initially proposed by Ni et al. [5]. In this technique, each pixel value is altered at most by 1, so this assures imperceptibility in the watermarked image. It is further observed that the embedding capacity of the histogram-based method is limited and is image dependent. Instead, if the difference value between adjacent pixels is chosen to represent the histogram, it can effectively increase the peak value and more amount of information can be embedded into the image without sacrificing the quality of the output image. There are many modifications suggested in the literature in the basic approach primarily to increase the hiding capacity. One method by Jung and Ko [6] suggests an improved histogram modification-based reversible data hiding technique. In this algorithm, human visual system characteristics are exploited to decide the data embedding level for each pixel. Another variation by Arabzadeh et al. [7] suggests generalization of the well-known histogram shifting method for reversible watermarking. A new reversible image authentication technique [8] based on watermarking utilizes histogram characteristics of the difference image and modifies pixel values slightly to embed more data than other lossless data hiding algorithms. Another work [9] proposes a blind reversible watermarking approach for medical images based on histogram shifting in a wavelet domain. Recent work by Cao and Zhou [10] proposes rhombus prediction model and difference histogram shifting idea for images. Various other researchers have tried to improve the embedding capacity of the basic histogram shift technique [11–14]. All the methods mentioned before increase computational complexity of the histogram shift method. Two of the most important requirements for a reversible watermark are the imperceptibility of the watermark and the hiding capacity. Both should be achieved with as less complexity as possible. Therefore, to design a reversible watermarking technique with increased hiding capacity and to produce output image with acceptable imperceptibility with minimal increase in complexity are the contributions of this paper. All the methods mentioned before increase computational complexity of the histogram shift method. In this paper, two new techniques are proposed which modify the histogram shift method to improve the hiding capacity. The first technique divides the image into nonoverlapping blocks and considers the histogram of each block. In each block, the watermark is embedded. This technique is a simple extension of the classic histogram shift method, but it provides an almost 10% increase in the embedding capacity. This technique takes full advantage of the pixel correlation in a small block of image. Not just that but by changing the size of the block, the required values of the embedding capacity and PSNR can be achieved. A large block size will improve PSNR while reducing capacity. It should be noted that the histogram shift technique discussed here affects all the pixels in the watermarked medical image. Medical professionals remain sceptical about allowing possible alteration of all pixels in the medical image irrespective of the watermarking technique used. This scepticism motivates researchers to consider RONI (region of noninterest) that are of no or little interest to doctors or medical professionals. In the second technique, first, the RONI is separated from the medical image and the watermark is embedded in this region. This region can have irregular shape. The algorithm will embed the watermark in this region of noninterest. Degradation caused with this type of embedding affects the only RONI by keeping intact the region of interest. The PSNR values achieved with this technique are also comparable to the existing methods.

The next section gives the details about the implemented methods.  Section 3 demonstrates the results for the proposed methods and Section 4 is the conclusion.

## 2. Methodology

In this section, the novel approaches for reversible watermarking are discussed. Firstly, the classical histogram shift method is revisited. Then, the block-wise embedding technique for the watermarking is explained. While embedding watermark in the medical image, care needs to be taken in order that the region of interest in the image is not tempered. To achieve this, the third method is proposed where the region of noninterest is separated from the image and the watermark is embedded only in the region of noninterest. The performance evaluation parameters used for determining the imperceptibility and the quality are the peak signal-to-noise ratio (PSNR), the mean square error (MSE) [15], and MSSIM [16]. Another parameter for performance measurement is the size of the watermark that can be embedded in the original medical image. This is the watermarking capacity. Let *I* denote the original medical image; whereas *I*_*w*_ denotes the watermarked image then MSE is calculated according to the following:

(1) $$\begin{array}{c} MSE\left(I,I_{w}\right)=\frac{1}{M\times N}\overset{M}{\underset{i=1}{\sum }}\overset{N}{\underset{j=1}{\sum }}{\left(I\left(i,j\right)-I_{w}\left(i,j\right)\right)}^{2}. \end{array}$$

Mean square error estimates the average of the square of the errors. The lower the value of MSE, the lower the errors and the higher the imperceptibility. *M* × *N* is the size of the image. PSNR is calculated based on the following:

(2) $$\begin{array}{c} PSNR\left(I,I_{w}\right)=10*\text{log}\left(\frac{2^{8}-1}{MSE}\right). \end{array}$$

Higher PSNR indicates higher imperceptibility of the watermark in the image.

In the last three decades, a great deal of effort has gone into the development of quality assessment methods that take advantage of known characteristics of the human visual system (HVS). Here, a measure of structural similarity (SSIM) that compares local patterns of pixel intensities that have been normalized for luminance and contrast is used to access the similarity between the original and the watermarked image. The structural similarity (SSIM) index [16] is based on the computation of three terms, namely, the luminance term, the contrast term, and the structural term. The overall index is a multiplicative combination of the three terms. For two images *x* and *y*,

(3) $$\begin{array}{c} SSIM\left(x,y\right)={\left[l\left(x,y\right)\right]}^{\alpha }\cdot {\left[c\left(x,y\right)\right]}^{\beta }\cdot {\left[s\left(x,y\right)\right]}^{\gamma }, \end{array}$$

where

(4) $$\begin{array}{c} l\left(x,y\right)=\frac{\;2\mu_{x}\mu_{y+C_{1}}}{\mu_{x}^{2}+\mu_{y}^{2}+C_{1}},\\ c\left(x,y\right)=\frac{2\sigma_{x}\sigma_{y}+C_{2}}{\sigma_{x}^{2}+\sigma_{y}^{2}+C_{2}},\\ s\left(x,y\right)=\frac{2\sigma_{xy+}C_{3}}{\sigma_{x}\sigma_{y+C_{3}}}, \end{array}$$

where *μ*_*x*_, *μ*_*y*_, *σ*_*x*_, *σ*_*y*_, and *σ*_*xy*_ are the local means, standard deviations, and cross-covariance for images *x*, *y*. If *α* = *β* = *γ* = 1 (the default for exponents) and *C*_3_ = *C*_2_/2 (default selection of *C*_3_), the index simplifies to the following:

(5) $$\begin{array}{c} SSIM\left(x,y\right)=\frac{\left(\;2\mu_{x}\mu_{y}+\right)\left(2\sigma_{xy}+C_{2}\right)}{\left(\mu_{x}^{2}+\mu_{y}^{2}+C_{1}\right)\left(\sigma_{x}^{2}+\sigma_{y}^{2}+C_{2}\right)}. \end{array}$$

Generally, SSIM is calculated for blocks of the image and then mean SSIM (MSSIM) is used as the performance parameter.

### 2.1. The Classical Histogram Shift Method and Its Limitations

In the general histogram shift method proposed by Ni et al. [5], data embedding is performed by shifting the one-dimensional histogram of the image. Consider image *I* with gray scale values ranging from 0 to 255. Consider *p* the gray scale value in image *I* which is the most frequently occurring gray value and *c* be the maximum count of the grayscale value *p*. The histogram is shifted to the right by one bin each according to the following rules and shifted image *I*_shift_ is formed:

(6) $$\begin{array}{c} I_{shift}=I_{i,j}\;if\;I_{i,j}=<p\;or\;if\;I_{i,j}>=253,\\ I_{shift}=I_{i,j}+1\;if\;I_{i,j}>p\;and\;I_{i,j}<253. \end{array}$$

Consider *m* the pixel value of the watermark image which is either “1” or “0.” The watermarked image $\tilde{I}$ is formed according to following conditions:

(7) $$\begin{array}{c} \tilde{I}=I_{shift}\;if\;I_{shift}=p\;and\;m=0,\\ \tilde{I}=I_{shift}+1\;if\;I_{shift}=p\;and\;m=1. \end{array}$$

**Figure 1:**
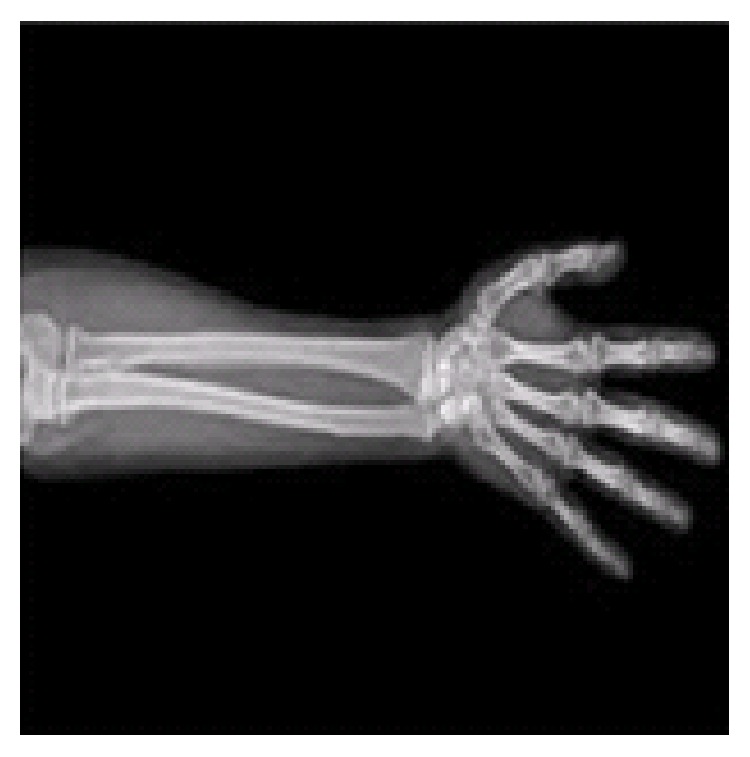
Original medical image.

**Figure 2:**
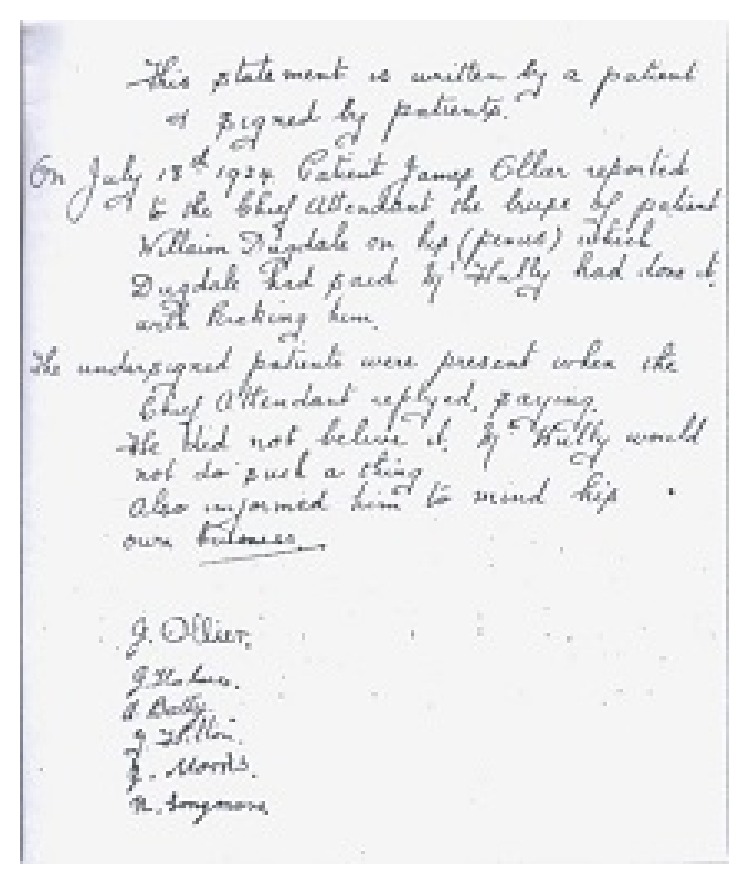
Watermark image.

To demonstrate the various techniques, different modality medical images are considered here. The image sizes ranged from 544 × 304 to 1002 × 1132.  Figure 1 shows the medical image, X-ray image used which is of size 900 × 854.  Figure 2 shows the watermark image used which is of size 256 × 256. This research considers the patient's medical report in the form of an image which is a generalized consideration. So, it is evident that all the discussed algorithms can be performed for text data as well. The watermark has the patient's information and is a black and white image.

**Figure 3:**
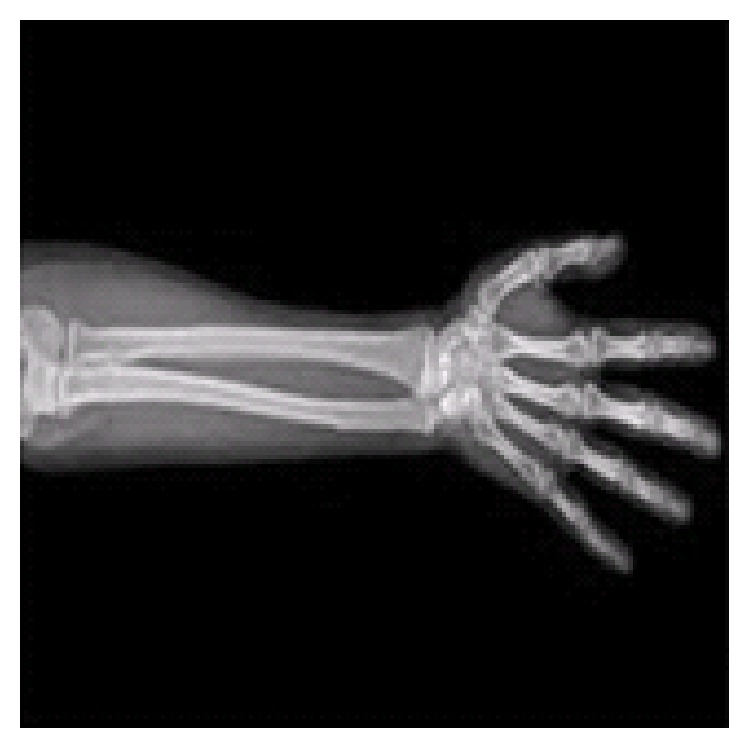
Watermarked image.

The results were obtained with the help of MATLAB. The image in Figure 3 is a watermarked image using the classical histogram shift technique. The capacity with this method for this image is 44,417 bits. This is around 6% of the total no of pixels. The watermarked image confirms that the watermark is imperceptible. Moreover, the MSE is 0.9831 and the PSNR is 47.92 which are much better than the accepted values. The most important advantage of the histogram shift technique is its simplicity in implementation. The computations required are only the calculations of the histogram which are very simple and fast. It needs to be noted that the capacity changes from image to image. The capacity of the watermark is dependent on the type of the image and the image pixel distribution. Overall, it can be observed that the capacity using the histogram shift technique is limited for medical images. To address this shortcoming of the histogram shift technique, two modifications are suggested in this paper. These proposed techniques increase the embedding capacity but at the same time, they keep the calculations simple. These two novel techniques are discussed in the following subsections.

### 2.2. Block-Wise Watermark Embedding [17]

To improve the limited watermarking capacity provided by the classical histogram shift technique, here, a new method is proposed. In this technique, the first cover medical image is divided into nonoverlapping blocks of equal size. The blocks are chosen to be nonoverlapping so a pixel will not be embedded with multiple bits. The histogram is calculated for each block and the maximum count greyscale value is noted along with the maximum count. Then, the watermark is embedded in each block. Each block can accommodate watermark bits equal to the maximum count in that block. For the image shown in Figure 1, the embedding capacity increases from 44,417 bits for the classical histogram shift method to 48,164 bits with this technique using the block size 4 × 4. This is almost a 10% increase in the hiding capacity. Still, the PSNR is fairly high. Similar results are observed for other medical images also. This technique exploits the correlation between the image pixels. As the histogram is plotted for smaller blocks of the image, the histogram is peakier. This increases the overall embedding capacity for the cover image. The watermark as well as the cover image can be retrieved completely.

**Figure 4:**
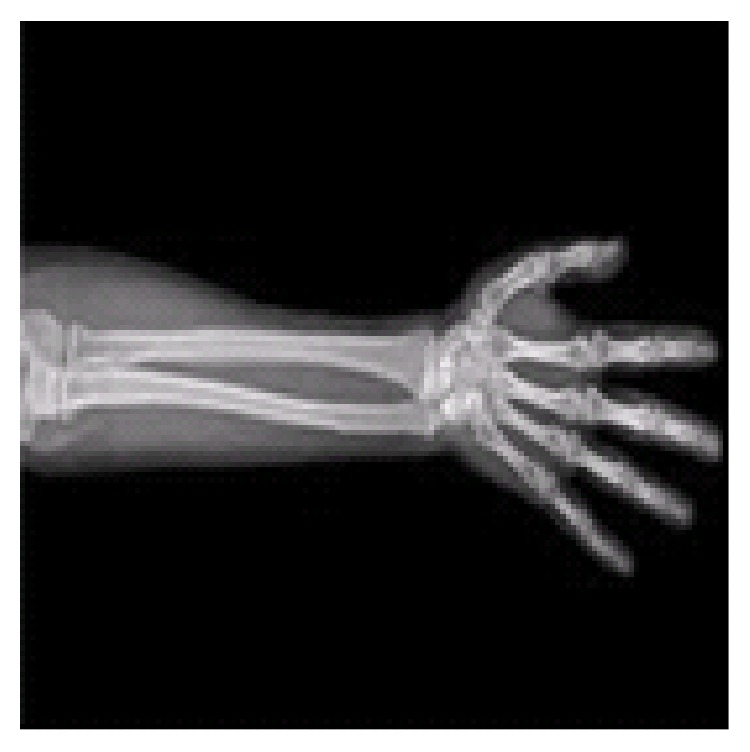
Watermarked image for block size 4 × 4.

Further, the algorithm is tested for varied block sizes like 4 × 4, 8 × 8, and 16 × 16. It is observed that as the block size increases, the embedding capacity decreases as excepted. In this technique, though the histogram needs to be calculated for each block, the overall complexity of the algorithm is still simpler compared to that in other reversible watermarking algorithms.  Figure 4 shows the watermarked image using a 4 × 4 block for the cover image in Figure 1.

### 2.3. Embedding in the Region of Noninterest (RONI)

**Figure 5:**
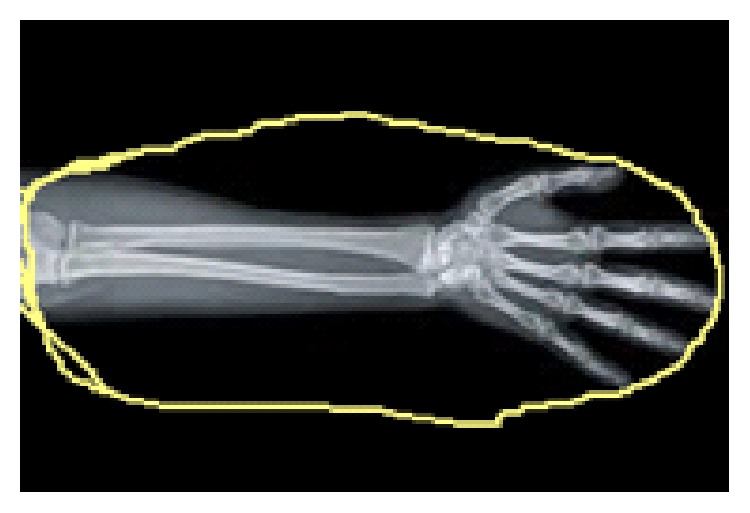
Image with the region of interest (ROI).

While hiding the watermark in a medical image, it is very important that the region of interest in the image should be kept unaltered. This is very important for the medical conclusions [18]. If the region of interest (ROI) in the medical image is modified, this may cause wrong diagnosis and could be fatal. When the histogram shift algorithm is used for embedding data in medical images, it is bound to change the contents of the region of interest. To prevail over this flaw, here, a novel idea is proposed where in the watermark is embedded in only in region of noninterest (RONI). Hence, this technique completely preserves the region of interest and the medical conclusions are unaffected. In this technique, first, the region of noninterest is separated from the medical image. In Figure 5, the circled region is the region of interest for this medical image which is the hand X-ray.

**Figure 6:**
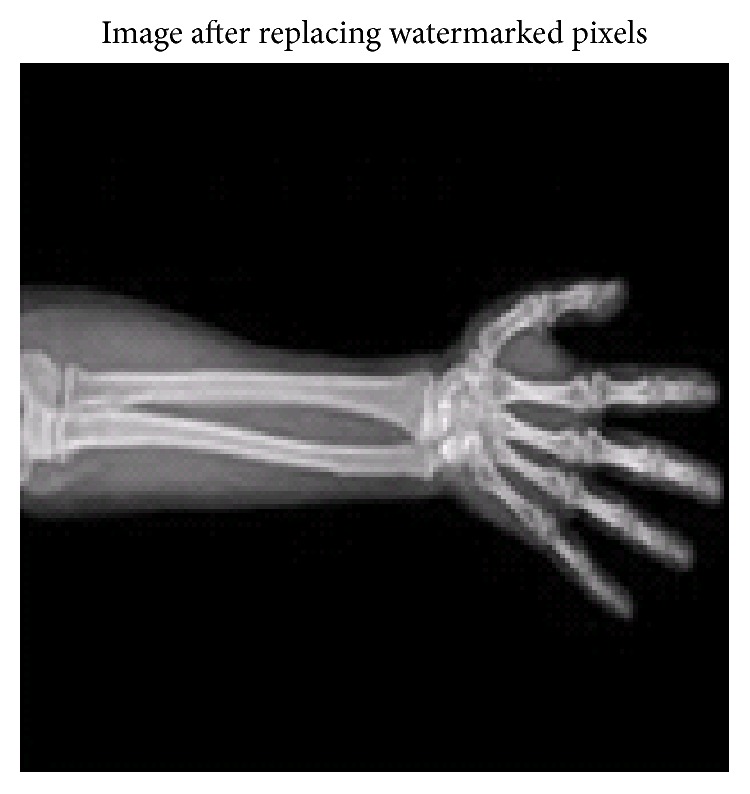
Watermark embedded in the RONI.

This ROI is separated using Out's method [19]. There are various techniques documented for separating the region of interest for medical images. Here, we have chosen Otsu's method which uses adaptive thresholding to separate the ROI from the RONI. This method is tested for various medical image modalities and gives satisfactory results. The method is implemented using MATLAB command which chooses a proper threshold for the image and converts it to a binary image. Using this binary image, the region of noninterest can be identified and the watermark is selectively embedded in this RONI. This RONI will have irregular shape. Once the region of interest is separated, the histogram of the RONI is plotted and the classical histogram shift technique is used to embed the watermark. After separation of the ROI, the remaining image is more correlated and hence has peakier histogram. Hence, in this technique, the embedding capacity is increased. Not only as the capacity improves but also as the watermark is embedded only in the RONI, the medical information in the cover image is kept intact. Moreover, the histogram shift method alters the pixel values closer to the peak. So, at the receiver end, again, the RONI can be obtained using Otsu's method. So, no extra information needs to be transmitted with the payload. For the image shown in Figure 1, the embedding capacity using this technique is 44,494 bits which is improved over the classical histogram technique. MSE and PSNR values obtained are 0.6013 and 49.99, respectively, which are fairly satisfactory. The MSSIM is 0.9386 which is closer to 1.  Figure 6 shows the watermarked image where the watermark is embedded in the RONI for the cover image in Figure 1.

## 3. Results and Discussion

All the reversible watermarking techniques explained in the previous section are tried on various types of medical images. The algorithms are implemented using MATLAB.

The first implemented method performs the watermark embedding based on the classical histogram shift technique.  Table 1 compiles the results for this method for three medical images. Cover1 image is the image shown in Figure 1(). The table contains the image sizes and the embedding capacity in bits for each image. Also, it records the MSE and PSNR values for the watermarked images for the three considered medical images. The watermark image used for embedding is a black and white image of size 256 × 256. The hiding capacity of each image is different.

For a given image, the watermark image is resized to the maximum hiding capacity for that image and the watermark is embedded using the histogram shift method. It can be concluded that with this technique, the PSNR value achieved is very high. So, the method is imperceptible. The calculations are very simple—only those required for plotting histogram of the image. So, the method is easy to implement.

The classical histogram shift technique has limited hiding capacity which changes from image to image. To improve the embedding capacity, a novel variant of the classical histogram shift technique is proposed in Section 2.2. In this technique, the cover image is divided into nonoverlapping blocks of the same size. While implementing this technique, the block size is varied from 4 × 4, 8 × 8 and 16 × 16. In each block, the maximum occurring gray level is noted and the pixel count is also noted. Each block has a hiding capacity same as the maximum count for that block. The watermark is embedded in each block. The results are found for the three images; Cover1, Cover2, and Cover3. It can be seen that the hiding capacity is increased when the watermark is embedded block wise. Also, as the block size is increased, the hiding capacity decreases.  Table 2 indicates these results. Columns 3, 4, and 5 show the hiding capacity for different block sizes for the three images. The capacity reduces with an increasing block size.  Table 3 records the MSE, PSNR, and MSSIM values for the three images for different block sizes. High PSNR value and MSSIM value confirm the imperceptibility of this algorithm which are important criteria for watermarking medical images. The algorithm is simple to implement so the advantage of simplicity is still maintained.

In the new technique discussed in Section 2.3, the RONI of the image is separated first. Then, histogram for this separated region is plotted and the watermark is embedded in this region. This technique has an important advantage over the previous two methods. It keeps the ROI of the medical image intact. So, the watermark embedding does not disturb the medical implications. Referring Table 2, the last column indicates the hiding capacity with this technique for the three images. There is an improvement in the capacity compared to classical histogram shift method.  Table 4 lists the MSE, PSNR, and MSSIM values for this technique. Here, though the capacity is improved, MSE is low whereas PSNR and MSSIM are higher. Also, the distortion in this method is only in the RONI. The following discussion brings out the significance of the techniques presented in the foregone text.

Most of the watermarking techniques modify the host image and thereby distort it while embedding the watermark. In many applications, these distortions or loss of fidelity is acceptable if the original image and the watermarked image are perceptually equivalent. On the contrary, images in some typical applications have stringent constraints on the image fidelity and thus, distortions during watermarking are not acceptable. Some of the application areas wherein images have stringent constraints on the image fidelity are medical images, military surveillance images, spy-satellite images, and legal document images. The reversible watermarking techniques, as discussed in this paper, are recommended for such applications mainly due to the lossless property of watermarking. It may be noted that the techniques presented in this paper give special emphasis on the reversibility property. In principle, these techniques can be applied to all types of images; however, its effectiveness gets highlighted in the applications where reversibility of the image is imperative.

Both the novel techniques proposed are reversible, so they can extract the watermark as well as the original medical image accurately.

## 4. Conclusion

Medical data exchange on the communication channel needs to be secured and authenticated. This can be achieved by means of watermarking the patient's data in the medical image itself. These watermarking techniques have to be carefully chosen as the data embedded should not hinder the vital medical information. Also, the techniques chosen should be reversible, that is, the cover medical image as well as the watermark should be extracted accurately. In this research work, the classical histogram technique is discussed with its application for medical images. To overcome the limited available watermarking capacity of this algorithm, two innovative techniques are suggested. For the block-wise embedding technique, it is observed that with a smaller block size like 4 × 4, higher embedding capacity is accomplished compared to larger block sizes like 8 × 8 or 16 × 16. The quantitative results indicate high imperceptibly with this technique. The second technique embeds watermark only in the region of noninterest. This technique provides lesser improvement in the hiding capacity compared to the block-wise technique. But in case of critical medical images where small distortion in the region of interest cannot be tolerated, this technique changes only the region of noninterest. Moreover, both the techniques preserve the simplicity of the original method. These innovative variants are well-suited techniques for medical image watermarking with higher capacity and better imperceptibility. These techniques are more suitable and beneficial for the applications that put stringent restriction on the image distortion.

## Figures and Tables

**Table 1:** Results of the classical histogram shift technique.

Images	Image size	Hiding capacity	MSE	PSNR
Cover1	900 × 854	44,417	0.9831	47.92
Cover2	1002 × 1132	36,947	1.086	47.49
Cover3	544 × 304	37,651	1.076	47.53

**Table 2:** Comparison of the hiding capacity for novel variants with the classical histogram shift technique.

Image	Image size	Hiding capacity in bits (histogram shift)	Block-wise histogram shift in bits (4 × 4)	Block-wise histogram shift in bits (8 × 8)	Block-wise histogram shift in bits (16 × 16)	Embedding in non-ROI in bits
Cover1	900 × 854	44,417	48,164	46,201	45,367	44,494
Cover2	1002 × 1132	36,947	42,258	39,567	38,253	37,103
Cover3	544 × 304	37,651	43,784	41,211	39,499	37,862

**Table 3:** MSE, PSNR, and MSSIM values for the block-wise embedding technique.

Images	Block size 4 × 4			Block size 8 × 8			Block size 16 × 16		
	MSE	PSNR	SSIM	MSE	PSNR	SSIM	MSE	PSNR	SSIM
Cover1	0.8266	48.68	0.9149	0.8481	48.56	0.9146	0.8831	48.39	0.9148
Cover2	0.7822	49.02	0.9273	0.7962	48.96	0.9297	0.8187	48.82	0.9271
Cover3	0.7668	49.28	0.9325	0.7766	49.23	0.9321	0.8022	49.08	0.9317

**Table 4:** MSE, PSNR, and MSSIM values for the technique using non-ROI.

Image name	MSE	PSNR	MSSIM
Cover1	0.6013	49.99	0.9386
Cover2	0.3431	52.63	0.9659
Cover3	0.5292	50.72	0.9476

## References

[1] Garg V., Brewer J. (2011). Telemedicine security: a systematic review. *Journal of Diabetes Science and Technology*.

[2] Coatrieux G., Lecornu L., Sankur B., Roux C. A review of image watermarking applications in healthcare.

[3] Zain J., Clarke M. Security in telemedicine: issues in watermarking medical images.

[4] Rey C., Dugelay J.-L. (2002). A survey of watermarking algorithms for image authentication. *EURASIP Journal on Applied Signal Processing*.

[5] Ni Z., Shi Y.-Q., Ansari N., Su W. (2006). Reversible data hiding. *IEEE Transactions on Circuits and Systems for Video Technology*.

[6] Jung S.-W., Ko S.-J. (2011). A new histogram modification based reversible data hiding algorithm considering the human visual system. *IEEE Signal Processing Letters*.

[7] Arabzadeh M., Helfroush M. S., Danyali H., Kasiri K. Reversible watermarking based on generalized histogram shifting.

[8] Gui X., Li X., Yang B. Efficient reversible data hiding based on two-dimensional pixel-intensity-histogram modification.

[9] Golpîra H., Danyali H. Reversible blind watermarking for medical images based on wavelet histogram shifting.

[10] Cao L., Zhou H. (2016). A new reversible date-hiding algorithm for encrypted images. *Mathematical Problems in Engineering*.

[11] Vinoth Kumar C., Natarajan V., Bhogadi D. High capacity reversible data hiding based on histogram shifting for medical images.

[12] Hazarathaiah K. (2014). Reversible data hiding using histogram shifting technique. *International Journal of Computer Science and Mobile Computing*.

[13] Nguyen T. H., Duong D. M. Reversible medical image watermarking technique based on choosing threshold values in histogram shifting.

[14] Nemade H. S., Kelkar V. Reversible watermarking for colored medical images using histogram shifting method.

[15] Fallahpour M., Megias D., Ghanbari M. High capacity, reversible data hiding in medical images.

[16] Wang Z., Bovik A. C., Sheikh H. R., Simoncelli E. P. (2004). Image quality assessment: from error visibility to structural similarity. *IEEE Transactions on Image Processing*.

[17] Kelkar V., Nemade H. Reversible watermarking in medical images using histogram shifting method with improved security and embedding capacity.

[18] Memon N. A., Gilani S. A. M. NROI watermarking of medical images for content authentication.

[19] Otsu N. (1979). A threshold selection method from gray-level histograms. *IEEE Transactions on Systems, Man, and Cybernetics*.

